# Biophysical Characterization of Shrimp Hemocyanins: Stability and Emerging Biotechnological Applications

**DOI:** 10.3390/biom15050675

**Published:** 2025-05-06

**Authors:** Lierge Ramos, Claudemir O. Souza, Ísis Sebastião, Giovana Bertini, Francisco Adriano de Oliveira Carvalho, Regildo Márcio Gonçalves da Silva, Edson Miguel Vilanculo, Julianne Soares Pereira, Patrícia Soares Santiago

**Affiliations:** 1 Institute of Chemistry, São Paulo State University Júlio de Mesquita Filho (UNESP), Araraquara 14800-900, SP, Brazil; lrg.ramos@hotmail.com (L.R.); claudemir.isouza@gmail.com (C.O.S.); isisebastiao@gmail.com (Í.S.);; 2 Faculty of Agricultural Sciences of Vale da Ribeira, Registro Campus, São Paulo State University (UNESP), Registro 11900-000, SP, Brazil; giovana.bertini@unesp.br; 3 Institute for Advance Studies of the Sea (IEAMar), São Paulo State University (UNESP), São Vicente 11350-011, SP, Brazil; 4 Institute of Exact Sciences, Federal University of South and Southeast Pará (UNIFESSPA), Marabá 68507-590, PA, Brazil; adriano.carvalho@unifesspa.edu.br; 5 Postgraduate Program in Sciences and Biotechnology, Fluminense Federal University, Niterói 24020-140, RJ, Brazil; juliannesope@gmail.com

**Keywords:** crustaceans, arthropods, hemocyanin, bioactive peptides, spectroscopy analyses, therapeutic applications

## Abstract

Hemocyanins are oxygen-transporting proteins found in crustaceans and other arthropods, playing key roles in immune defense and metabolic regulation. Due to their stability and bioactive properties, Hcs have gained increasing interest in biotechnological and biomedical applications. However, detailed biophysical characterization is crucial to understanding their functional potential. In this study, the hemocyanin was extracted and purified from *Macrobrachium acanthurus* (HcMac) using ultracentrifugation and size-exclusion chromatography. The molecular mass of HcMac was determined by SDS-PAGE electrophoresis, MALDI-TOF mass spectrometry, and analytical ultracentrifugation. Spectroscopic analyses, including UV-Vis absorption, fluorescence emission, and light scattering intensity, were used to assess the structural stability of the compound under various pH conditions. HcMac was identified as a hexameric protein (~450 kDa) composed of monomeric subunits of 75 and 76 kDa. The protein maintained its oligomeric stability and oxygen-binding affinity in the pH range of 5.0–7.4. However, extreme pH conditions (below 4.4 and above 7.5) induced structural alterations, leading to dissociation and conformational changes, as evidenced by fluorescence emission and UV-Vis spectra. The isoelectric point was determined to be between pH 4.3 and 5.3, consistent with other crustacean HCs. These findings reinforce the structural robustness of HcMac and suggest its potential for biotechnological applications. The high stability of HcMac under physiological pH conditions indicates its suitability for biomedical research, including immunomodulatory and antimicrobial applications. Future studies integrating bioinformatics, proteomics, and immunological assays will be essential to explore the therapeutic potential of HcMac.

## 1. Introduction

### 1.1. General Overview of Hemocyanins in Crustaceans and Mollusks

Hemocyanin (Hc) is a protein found in crustaceans and other invertebrates, such as arthropods and mollusks, dissolved in hemolymph. Its main function is the transport of oxygen, in addition to playing an important role in the delivery of exogenous copper to sites of accumulation of resistant pigments [1]. Hc is a blue respiratory hemoprotein, its blue color being due to the presence of copper in its heme group (di-copper center). However, the molecular architecture of the aggregates and the size of the structural and functional units are different in arthropods and mollusks [2]. In arthropods, each subunit contains an active di-copper site, while in mollusks, each functional subunit contains a site that binds reversibly to oxygen [3]. This protein has been recognized as a multifunctional protein, performing several essential physiological functions, such as protein storage, osmotic regulation, ecdysone hormone transport, exoskeleton formation, and immune defense [1,4]. Hc constitutes > 90% of the total plasma protein of crustaceans [4]. It is found in concentrations greater than 100 mg mL^−1^ and stable in extreme temperatures ranging from −20 °C to 90 °C [2].

### 1.2. Essential Biological Functions, Emphasizing Oxygen Transport and Immune Defense

**Figure 1:**
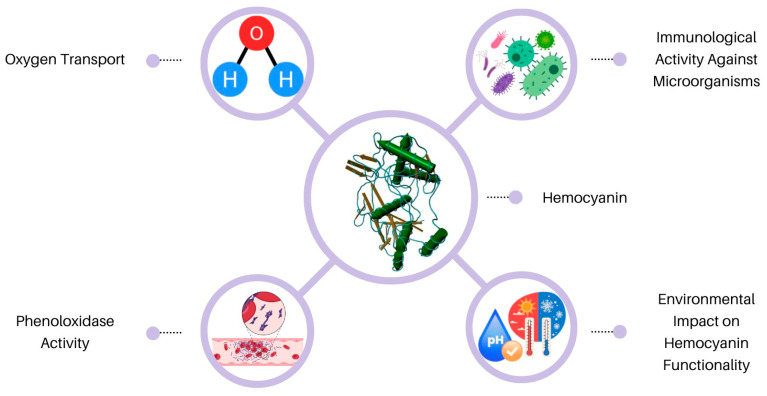
Functions and activities of hemocyanin in crustaceans: oxygen transport, phenoloxidase activity, immune response, and environmental impact (Adapted from [5,6,7]).

Hc is a multifunctional crustacean protein, playing critical roles in oxygen transport and immune response. It is responsible for facilitating oxygen transport within the respiratory system of these invertebrates, thereby contributing to respiratory homeostasis [5]. Several studies have demonstrated that Hc is an essential component of hemolymph, functioning as a key protein in osmotic regulation and storing crucial metabolic substances in crustaceans [6,7]. Figure 1 demonstrates the diverse functions of Hc, emphasizing its role in oxygen transport, its phenoloxidase (PO) activity, and its contribution to immune defense. Additionally, it highlights how environmental factors may influence the structural and functional stability of Hc, which is essential for its physiological and biotechnological applications [5,6,7].

Therefore, the expression of Hc in crustaceans provides them with immunological properties due to protein peptides that contribute to complementary innate immune responses, including antiviral, hemolytic, melanotic, and phenoloxidase (PO) activities [8].

#### Molecular Structure of Hemocyanins

Hc exhibits structural diversity due to genetic rearrangements and duplications, enabling its adaptation to different ecosystems [9]. Structural characterization studies have shown that arthropod Hcs aggregate into oligomeric complexes with a molecular mass (MM) of approximately 3.4 MDa [10]. These oligomers can exist as hexamers (1 × 6), dihexamers (2 × 6), or oligohexamers (4 × 6), with each hexamer composed of six identical or similar monomeric subunits. Each monomer contains 620–660 amino acid residues, has an MM of approximately 75 kDa [6,11,12], and is organized into three structural domains. Domains I and II are predominantly α-helical, while Domain III consists of irregular structures and β-sheets [13,14].

Hc adopts a cylindrical arrangement in mollusks, forming decameric or multi-decameric subunits ranging from 330 to 450 kDa. Each functional unit contains a copper-binding center essential for oxygen transport [14,15]. These functional units are sequentially arranged along polypeptide subunits via peptide linkages of 10–20 amino acids, forming a quaternary structure that can be readily visualized using transmission electron microscopy ( FEI Company, Hillsboro, OR, USA) [15,16].

Furthermore, Hc exhibits resistance to alkaline pH, characterized by binuclear copper atoms coordinated by three histidine residues. Despite structural similarities between arthropods and mollusks, the degree of conservation in the structural organization of Hc differs significantly between these two groups [14,17].

Given its structural diversity and biological significance, the crystalline structure of Hc can be analyzed using X-ray crystallography. However, to obtain high-purity protein for structural studies, hemolymph must first undergo ultracentrifugation to isolate the protein fraction as a pellet, followed by spectrophotometric methods for concentration estimation [6,14].

As previously mentioned, hemocyanins (Hcs), beyond their structural complexity, play essential roles in diverse biological processes, further emphasizing their functional versatility in invertebrate physiology [6,7].

### 1.3. Context of the Emerging Biotechnological Importance of Hemocyanins

The bioactivities observed in invertebrates (mollusks and arthropods), particularly crustaceans, are largely dependent on glycoproteins. Their absence can lead to metabolic disorders, including diabetes, and increased susceptibility to viral and microbial infections, highlighting their role in immune system regulation [18].

Hcs exhibit antimicrobial properties due to their enzymatic PO activity, which is activated under stress conditions and plays a key role in immune defense [1,19,20]. These properties make Hcs valuable for biotechnological applications, as they serve as a source of antibacterial, antifungal, antiviral peptides, and show potential antitumor activity [10,21,22,23].

Understanding the molecular structure and stability of Hcs is essential for therapeutic applications to ensure their efficacy and quality as glycoproteins [24]. Several studies have reinforced the biotechnological relevance of Hcs due to their glycoprotein composition, which underlies their diverse biological functions and potential biomedical applications [8].

#### Biotechnological Applications

Given the structural complexity and bioactive properties of Hcs, recent advances in biotechnology and biomedicine have highlighted their potential for therapeutic and diagnostic applications. Notably, peptide-based strategies have emerged as promising disease prevention and treatment tools. For instance, Lyu et al. developed a peptide vaccine targeting the ADAMTS-7 protein, a key factor in atherosclerosis and post-injury neointimal hyperplasia [25]. This vaccine effectively reduced atherosclerotic plaque formation and attenuated neointimal hyperplasia in experimental models, paving the way for novel cardiovascular disease therapies.

Further supporting the biomedical relevance of invertebrate-derived proteins, Alinejad et al. [26] conducted a proteomic analysis of *Macrobrachium rosenbergii* hemocytes infected with the IHHNV virus. Identifying differentially expressed proteins in response to infection provides valuable insights for diagnostic marker development and therapeutic strategies in aquaculture. Such advances could enhance sustainability in aquaculture practices by improving disease monitoring and treatment.

Table 1 summarizes key studies that explore various biotechnological applications of marine-derived proteins. These include environmental biomarker assessments in *Procambarus clarkii* and *Carcinus maenas* [27], molecular evolution studies in *Homarus gammarus*, *Carcinus maenas*, and *Penaeus monodon* [28], and investigations into immunological adjuvants in *Litopenaeus vannamei* [29]. More recently, ref. [29] explored the potential of hemocyanins from *Helix aspersa*, *Helix lucorum*, and *Rapana venosa* in cancer therapy. Additionally, ref. [30] examined bioactive peptides from *Carcinus maenas*, further reinforcing the importance of invertebrate-derived proteins in biomedical research.

### 1.4. Justification for Choosing Macrobachium Acanthurus as the Species of Study

Building upon the biotechnological relevance of hemocyanins (Hcs), *Macrobrachium acanthurus* presents an interesting model for studying immune defense mechanisms. This shrimp belongs to a group of crustaceans that rely solely on innate immunity, lacking adaptive immune responses [33,34]. Despite inhabiting environments prone to viral and fungal infections, these organisms have evolved a robust immune system closely linked to their metabolic pathways [35].

For instance, infections caused by the white spot syndrome virus (WSSV) not only activate key immune signaling pathways, such as Toll, but also alter metabolic homeostasis, increasing fatty acid and amino acid levels in the gills and hepatopancreas while modulating proteins involved in sterol regulation. These metabolic shifts highlight the complex interplay between immunity and physiological adaptation.

Therefore, this study aimed to perform a comprehensive biophysical characterization of hemocyanin from *Macrobrachium acanthurus* (HcMac), focusing on its structural stability under various physicochemical conditions to support future research on its biotechnological and biomedical applications.

## 2. Materials and Methods

### 2.1. HcMac Extraction and Purification

To extract hemolymph from *Macrobrachium acanthurus*, a total of 60 specimens were utilized. Each animal was immersed in cold water (5 °C) for 3–5 min to induce anesthesia. Next, a 1.0 mL syringe containing roughly 300 μL of 100 mmol L^−1^ sodium citrate anticoagulant was carefully inserted into the pericardial region of the animal to prevent contamination from hepatopancreatic materials. The hemolymph was then centrifuged at 3000× *g* and 4 °C for 15 min to eliminate tissue and other potential impurities.

Dialysis was utilized to eliminate low-molecular-weight constituents by using a 30 kDa cut-off membrane. The process was performed at 4 °C for 12 h against Tris-HCl buffer (100 mmol L^−1^) supplemented with 20 mmol L^−1^ CaCl_2_ and maintained at pH 7.0 (storage buffer).

The hemolymph of *Macrobrachium acanthurus* was subjected to ultracentrifugation at 250,000× *g* and 4 °C for 5 h to isolate the high molecular weight HcMac. The HcMac was obtained as a pellet at the bottom of the tube, and the supernatant was subsequently discarded. The HcMac was resuspended in a storage buffer and stored at 4 °C. The concentration was determined using a UV-1800 SHIMADZU spectrophotometer (Shimadzu, Tokyo, Japan) with (SEC) the appropriate molar absorption coefficient value of Ɛ_278_ nm = 1.1 (mg/mL) cm^−1^ [36].

During the final purification step, fractions of the 2.0 mL HcMac stock underwent size exclusion chromatography (SEC) using a Hiload 16/600 Superdex 200 column. The column was equilibrated against Tris-HCl 100 mmol L^−1^ + CaCl_2_ 20 mmol L^−1^ buffer, pH 7.0, and coupled to the AKTA Pure L GE instrument (Thermo Fisher Scientific, Waltham, MA, USA) with a UV detector at 280 nm. Subsequently, all the fractions were concentrated using an Amicon–Millipore membrane with a 30 kDa mass cut-off to achieve a final 150 mg mL^−1^ concentration in a 4 mL volume. Finally, the purified protein was stored at 4 °C to support further studies. All reagents were obtained from Sigma-Aldrich (St. Louis, MO, USA).

### 2.2. One-Dimensional SDS-PAGE Electrophoresis Experiments

To evaluate the molecular mass of HcMac subunits, SDS-PAGE was performed using a 10% (*v*/*v*) acrylamide gel at pH 8.6. Samples were prepared with and without the reducing agent β-mercaptoethanol in a 1:1 (HcMac/reducer) ratio and applied to the gels at concentrations ranging from 0.03 to 0.1 mg mL^−1^. Electrophoresis was conducted at a constant voltage of 140 V in a buffer containing 25 mmol L^−1^ Tris-HCl and 192 mmol L^−1^ glycine at pH 6.8. The gels were stained with Coomassie Brilliant Blue R-250 and marked using the Bio-Rad Precision Plus Protein^TM^ standard (Bio-Rad, Hercules, CA, USA).

### 2.3. MALDI-TOF-MS Mass Spectrometry Experiments

To prepare for MALDI-TOF-MS analysis, the purified HcMac underwent extensive dialysis against a 5.0 mmol L^−1^ sodium phosphate buffer to eliminate excess salt from the samples. The HcMac was diluted in the same dialysis buffer to a 1.0–3.0 mg mL^−1^ concentration range. Synaptic acid, β-mercaptoethanol, cytochrome *c* (bovine), and bovine serum albumin (BSA) were obtained from Aldrich and Sigma (Sigma-Aldrich, St. Louis, MO, USA). The laser desorption matrix material, Sinapinic acid (SA) (Sigma-Aldrich, St. Louis, MO, USA), was dissolved in a mixture of 0.5% trifluoroacetic acid and 50% acetonitrile/water. SA, a derivative of cinnamic acid, was selected as the matrix due to its well-documented efficiency in the ionization of high-molecular-weight proteins in MALDI-TOF-MS analyses. SA has strong absorption in the UV range (~335–340 nm), making it particularly suitable for proteins such as hemocyanins, which have subunits with molecular masses in the 75–100 kDa range. Compared to other matrices, sinapinic acid promotes soft ionization, reducing fragmentation and improving the detection of intact protein subunits. This makes it an ideal choice for studies focused on structural characterization and molecular weight determination of large biomolecules.

For analysis, native HcMac samples were diluted with a saturated solution of SA in ratios of 1:5, 1:10, and 1:20 (*v*/*v*) as described by [37]. One microliter of the matrix/sample mixture was applied to each spot on the MALDI plate and analyzed in linear, positive ion mode on an Amersham Bioscience Etthan MALDI-TOF mass spectrophotometer using an accelerating voltage of 20 kV. Each spot was analyzed twice, and the composite spectrum was generated by accumulating approximately 200 laser shots in total. The resulting spectrum was analyzed using Origin 8.5 software. The instrument was calibrated using standards such as BSA and cytochrome c. Reported molecular masses were calculated as an average of values obtained for mono- and di-protonated molecular species from several individual experiments.

### 2.4. Analytical Ultracentrifugation Analysis (AUC)

At the National Synchrotron Light Laboratory (LNLS) in Campinas, SP, the AUC experiments were conducted using a Beckman Optima XL-A analytical ultracentrifuge (Beckman Coulter, Inc., Brea, CA, USA). Sedimentation velocity (SV) experiments for HcMac were performed at 100, 200, and 300 μg mL^−1^ concentrations. The samples underwent a 48 h dialysis process against Tris-HCl 100 mmol L^−1^ buffer, containing NaCl 50 mmol L^−1^, at pH 7.0. During this time, the dialysis solution was replaced three times, and the final solution was used as the reference for all AUC cell experiments.

Protein dilutions were prepared two hours before the experiments using the most recent dialysis buffer. SV measurements were obtained using an An60Ti rotor with a rotational speed range of 15,000 to 40,000 rpm at 20 °C. Data acquisition was performed by monitoring the absorbance at 280 nm (aromatic amino acid region) at 7 min intervals.

SEDFIT version 14.1 was used to analyze the experimental AUC data obtained from SV experiments. The software was employed to fit sedimentation coefficient distribution functions, c(S), and molecular mass distribution functions, c(M), using the “Continuous c(S) Distribution model” [38]. During the analysis, the value of Vbar was held constant at 0.733 mL g^−1^, while the frictional ratio (*f*/*f*_0_) was the regularization parameter and remained unrestricted. The Sednterp program [5] was used to obtain input parameters such as buffer viscosity (*η*) and density (*ρ*). The values of s* from the c(S) curves fits were corrected for standard conditions (water solvent and 20 °C), and linear regression extrapolation was performed for conditions approaching infinitely dilute concentrations, i.e., close to 0 mg mL^−1^ protein. The sedimentation coefficient and molecular masses were determined at the maximum of the Gaussian of c(S) and c(M), respectively. A degree of confidence of *p* = 0.85 was set for the analyses [6].

### 2.5. Spectroscopic Analysis

The spectroscopic measurements were conducted at 25 °C in acetate–phosphate–borate buffer with a concentration of 30 mmol L^−1^, with the pH varying between 3.5 and 10.5. The final concentration of HcMac was 1.6 mg mL^−1^, and the pH of each sample was confirmed after adding protein to the buffer. Spectra were initially collected at 1, 3, 6, and 24 h after sample preparation, but no significant changes were observed after 3 h of equilibration. Therefore, all spectra included in this study were obtained after a 3 h equilibrium period.

Spectroscopic measurements were carried out using a quartz cuvette with an optical path length of 1.0 cm. Optical absorption measurements in the UV-Vis region were conducted on a SHIMADZU UV-1800 spectrophotometer (Shimadzu, Tokyo, Japan) in the 700–250 nm range. Static fluorescence emission and light scattering intensity (LSI) measurements were obtained on a SHIMADZU RF-6000 spectrofluorimeter (Tokyo, Japan). Fluorescence emission spectra were acquired by exciting at 295 nm and monitoring Tryptophan (Trp) emissions within the range of 305–450 nm, with an excitation slit width of 3.0 nm and emission of 5.0 nm. LSI measurements were conducted at 350 nm excitation and emission wavelengths using excitation and emission slits of 3.0 nm and 5.0 nm, respectively.

## 3. Results

### 3.1. Purification

**Figure 2:**
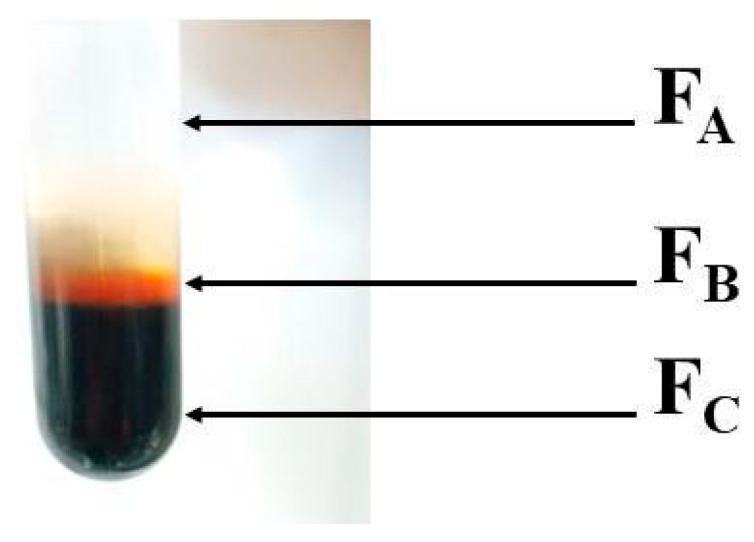
After the ultracentrifugation fractionation process was completed, a photo of a tube showed the separated components. The supernatant was labelled as (FA), the dark yellowish content identified as lipoprotein was labelled as (FB), and the dark blue sedimented HcMac (hemolymph from *Macrobrachium acanthurus)* was labelled as (FC), with a total protein concentration of 421.0 mg mL^−1^.

Following ultracentrifugation, three distinct fractions were identified and isolated within the tubes. The lightest part of the tube, designated as fraction A (FA), corresponded to the supernatant. The yellowish content present, identified as lipoprotein [39], was represented by fraction B (FB). The dark blue sedimented portion, fraction C (FC), corresponded to HcMac, as demonstrated in Figure 2.

SEC was recognized as a widely accepted protein purification technique, and it was demonstrated to be a reliable alternative for isolating and purifying Hcs and other high-molecular-weight proteins [36,39,40].

**Figure 3:**
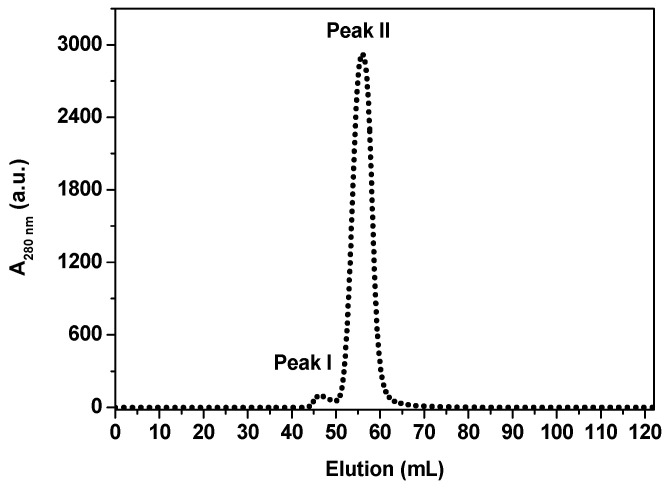
The size exclusion liquid chromatography elution profile of HcMac (hemocyanin from *Macrobrachium acanthurus)* (50.0 mg mL^−1^) was obtained using a Hiload 16/600 Superdex 200 PG column equilibrated with Tris-HCl 100 mmol L^−1^ and CaCl_2_ 20 mmol L^−1^ buffers at pH 7.0 and 25 °C, with a flow rate of 1.5 mL min^−1^.

Two peaks were observed in the chromatogram of HcMac (Fc Fraction shown in Figure 2 and Figure 3), with Peak II being the predominant species in the solution. However, Peak II exhibited the characteristic spectrum and molecular mass pattern of Hcs, indicating that the protein had not been completely purified after ultracentrifugation. Consequently, Peak II was identified as HcMac and was employed in subsequent studies.

### 3.2. One-Dimensional SDS-PAGE Electrophoresis

**Figure 4:**
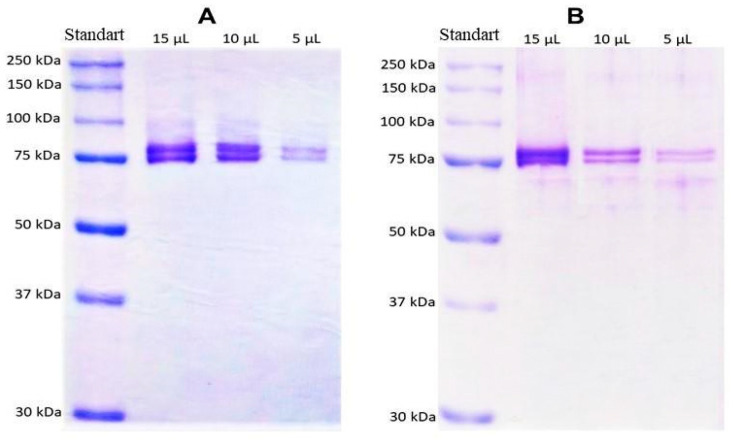
SDS-PAGE electrophoresis gel (10% acrylamide, *v*/*v*) of the fraction of Peak II (HcMac) from *Macrobrachium acanthurus* hemolymph after size exclusion chromatography (Figure 3). Different volumes were applied to the wells. (**A**) In the absence of the reducing agent β-mercaptoethanol; (**B**) in the presence of the reducing agent β-mercaptoethanol. Original Western blot images can be found in Figure S1.

The mass pattern of HcMac had remained unchanged in the presence of the reducing agent β-mercaptoethanol, as confirmed by SDS-PAGE analyses. Two distinct bands, with molecular masses near 75 and 76 kDa, had been observed in the gels. No significant differences had been detected between SDS-PAGE electrophoresis gels in the presence and absence of reducing agent β-mercaptoethanol, suggesting that the bands around 75 kDa had corresponded to the monomeric form of HcMac. At the same time, dimers or other oligomeric subunits had been absent under these denaturing conditions (Figure 4).

### 3.3. Analysis of MALDI-TOF-MS Spectra

**Figure 5:**
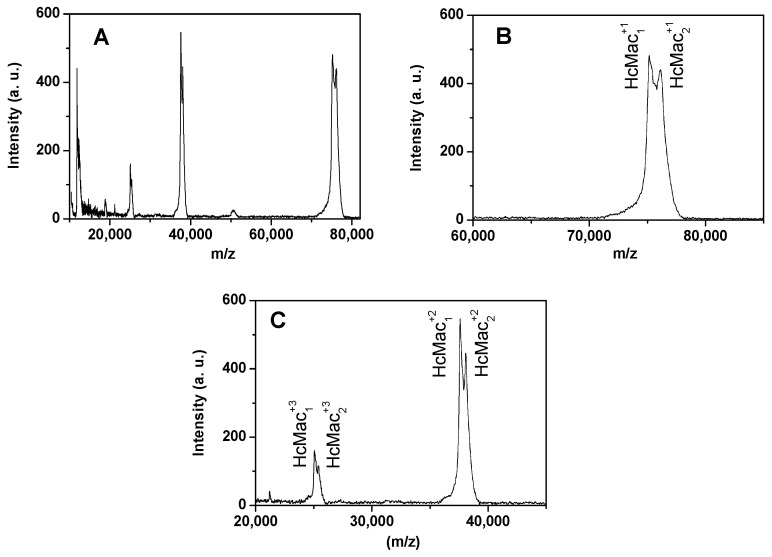
(**A**) MALDI-TOF-MS spectrum of hemocyanin from *Macrobrachium acanthurus* (HcMac), pH 7.0; (**B**) expanded mass region from 60,000 to 90,000 Da, for the mono-protonated monomeric chains; (**C**) expanded mass region from 20,000 to 45,000 Da, for the di- and tri-protonated monomeric chains.

Figure 5A displays the mass spectrum of HcMac at pH 7.0 using SA as the matrix in the positive ion mode. The peaks correspond to the ionization of the HcMac subunits, which displayed the most intense signals in the ranges between 35 and 40 kDa as well as 70 and 80 kDa.

The findings revealed that HcMac exhibited mono-protonated monomeric subunits (HcMac_1_^+1^ and HcMac_2_^+1^) with molecular weights of approximately 75 and 76 kDa, respectively (Figure 5B). Additionally, two di-protonated monomeric chains were detected at around 37 and 38 kDa, while two tri-protonated monomeric subunits appeared at approximately 25 kDa (Figure 5C).

### 3.4. Analysis of AUC Data

**Figure 6:**
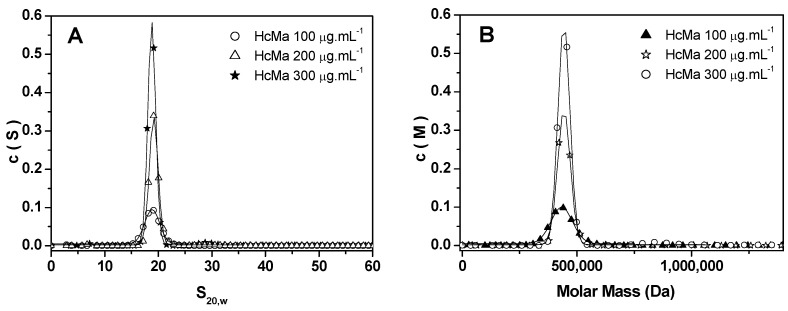
(**A**) Sedimentation coefficient curves were obtained for hemocyanin from *Macrobrachium acanthurus* (HcMac) at pH 7.0. (**B**) Continuous molecular mass distribution of HcMac at different concentrations in solution.

Figure 6A displays the c(S) distribution curves of HcMac at pH 7.0 for three different concentrations, revealing a single, sharp peak, indicating a high degree of homogeneity in the solution. The extrapolated sedimentation coefficient (s^0^_20w_) in aqueous solvent at 20 °C was determined to be 19S. Figure 6B shows the c(M) distribution curves corresponding to the same concentrations. By employing the s/D ratio, the molecular mass of HcMac was calculated to be approximately 450 kDa. This value is consistent with a hexameric assembly composed of six monomeric subunits. The MALDI-TOF MS analysis had previously revealed two distinct monomeric chains with molecular masses of 75 and 76 kDa. Together, these subunits form a 1 × 6 hexameric structure with a total molecular mass of 450 kDa, supporting the conclusion that HcMac exists predominantly as a stable and homogeneous hexamer in solution.

### 3.5. Spectroscopic Analysis of Native Hemocyanin from Macrobrachium Acanthurus (HcMac) as a Function of pH

**Figure 7:**
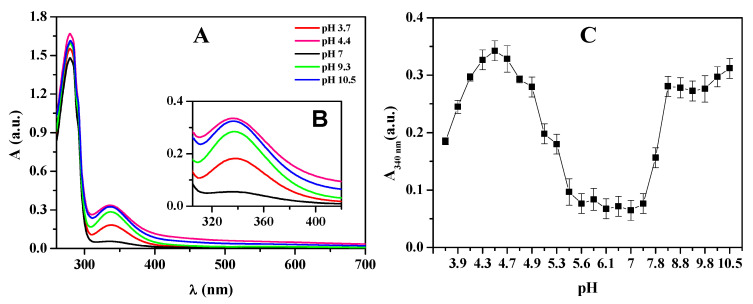
(**A**) Optical absorption spectra of hemocyanin from *Macrobrachium acanthurus* (1.6 mg mL^−1^) in acetate–phosphate–borate buffer 30 mmol L^−1^ in the pH range 3.7–10.5. (**B**) Inset from (**A**). (**C**) Absorption region at 340 nm of the spectra from (**A**). Error bars represent the standard deviation (±SD) of 5 different measurements, all performed at 25 °C.

The affinity of HcMac to oxygen was impacted by variations in pH, resulting in an amplified absorption spectrum band intensity at 340 nm (Figure 7) when the medium was either acidified or alkalized. The absorption intensity at 340 nm gradually increased as a function of raising pH, reaching its peak at pH 4.4. However, a decrease in absorption intensity at 340 nm was shown for pH values above 4.4. The 340 nm band remained consistent with low intensity within the pH range of 5.2 to 7.3. Above pH 7.3, there was a notable increase in absorption intensity, peaking at pH 8.0. However, the absorption intensity of the 340 nm band remained relatively constant between pH 8.0 and 10.5, exhibiting a slight tendency towards increased absorption intensity.

**Figure 8:**
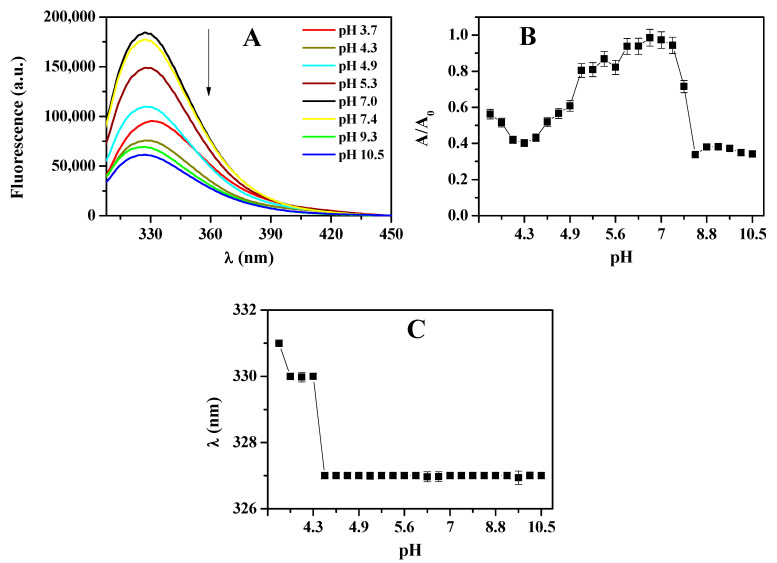
(**A**) Fluorescence emission spectra of hemocyanin from *Macrobrachium acanthurus* (1.6 mg mL^−1^), in acetate–phosphate–borate buffer 30 mmol L^−1^. (**B**) Total area of normalized fluorescence emission (A/A_0_) from the spectra shown in (**A**). (**C**) λ_max_ fluorescence emission from the spectra in (**A**). Error bars represent the standard deviation (±SD) of 5 different measurements, all performed at 25 °C.

The study also showed a decrease in fluorescence intensity for acidic and alkaline mediums compared to the neutral medium, pH 7.0 (Figure 8A). The fluorescence emission area normalized by the fluorescence emission area at pH 7.0 (Figure 8B) implied that HcMac exhibited higher stability in the pH range of 5.0 to 7.5. At pH below 5.0, fluorescence emission was continuously reduced until it reached pH 4.4. An increase in pH levels beyond 7.5 led to a drastic reduction in fluorescence, reaching a plateau at pH 8.3. These findings aligned with the analysis of the UV-Vis spectra, as shown in Figure 8C.

Based on the fluorescence emission results, it appeared that the denaturation process of HcMac was triggered at pH values lower than 4.4, as evidenced by the shift in the maximum wavelength (λ_max_) of fluorescence emission towards higher values (Figure 8C). These observations align with the UV-Vis analyses, indicating a decreased affinity for O_2_ at pH below 4.4 (Figure 8C).

**Figure 9:**
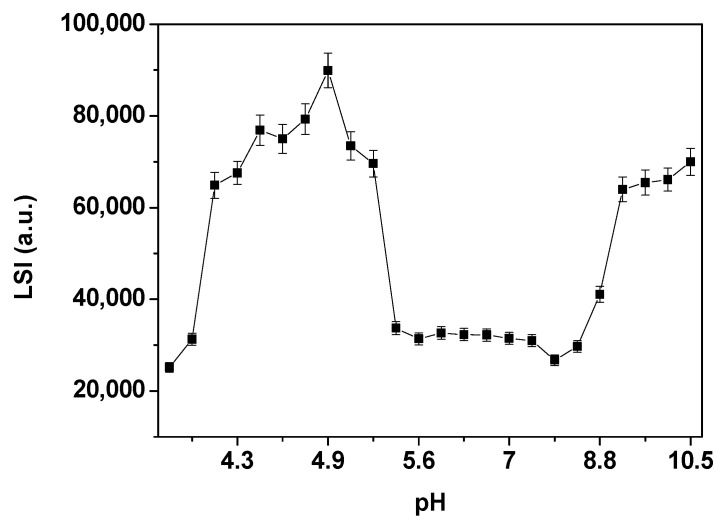
Light scattering intensity (LSI) of hemocyanin from *Macrobrachium acanthurus* (1.6 mg mL^−1^) in acetate–phosphate–borate buffer 30 mmol L^−1^ at different pH values. Error bars represent the standard deviation (±SD) of 5 different measurements, all performed at 25 °C.

The LSI measurements (Figure 9) showed that the LSI of HcMac remained relatively constant between pH 5.3 and 8.4, with two distinct plateaus of greater intensity. The first plateau appeared within the pH range of 4.2 to 5.3, which could be attributed to the isoelectric point (pI) of HcMac in the region with acidic pH values. The second plateau was observed between pH values 9.3 and 10.5 (Figure 9).

## 4. Discussion

The blue color of the hemolymph arises from the ligand–metal charge transfer (O_2_^2−^---Cu^2+^) in the oxygenated state of HcMac, which occurs due to the transition of Cu (I) to Cu (II) at the metal center. The HcMac has an absorption spectrum with an intense broadband at 340 nm and a molar absorptivity coefficient (Ɛ) of 20,000 M^−1^ cm^−1^ [2]. Thus, the F_C_ fraction, which exhibits an intense blue hue, likely has high concentrations of the HcMac form (Figure 2).

The ultracentrifugation process effectively fractionates shrimp hemolymph, but the FC chromatogram revealed that hemoprotein was not completely pure (Figure 3). Therefore, it was necessary to use SEC, which is a widely accepted technique for protein purification and has been demonstrated to be a reliable alternative for isolating and purifying Hcs and other high-molecular-weight proteins [40,41].

In Figure 4, the gels revealed two bands with molecular masses close to 75 and 76 kDa, consistent with previous reports on hemocyanins from other crustaceans [2,,36]. Hc subunits in the shrimps *Caridina multidentata* and *Atyopsis moluccensis* ranged between 77 and 87 kDa [11], while the lobsters *Astacus astacus* and *Cherax destructor* displayed different subunits between 71 and 98 kDa [42].

Studies on Hc from the shrimp *Macrobrachium rosenbergii* [40] identified four bands on a 7% acrylamide gel without a reducing agent: 50, 60, 114, and 325 kDa. However, when the proteins were reduced in SDS, it was revealed that the 50 and 60 kDa bands corresponded to monomers, the 114 kDa band represented a dimer composed of monomeric subunits of 56 and 58 kDa, and the 325 kDa band was a trimer consisting of subunits of 74, 76, and 78 kDa.

The gels showed no noticeable difference (Figure 4), indicating that the bands around 75 kDa correspond to the monomeric form of HcMac and that dimers or other subunit oligomers were not detected under these denaturing conditions. However, the stability of Hcs is known to rely on the aggregation of diverse subunits into various oligomeric forms to fulfil multiple biological functions [43,44].

The findings imply that the two monomeric subunits with masses of 75 and 76 kDa, MALDI-TOF-MS data (Figure 5), correspond to the two bands around 75 kDa observed in the acrylamide (10%) gels (Figure 4). Therefore, the outcomes suggest that HcMac comprises 75 and 76 kDa monomers, consistent with other shrimp Hcs [14,45,46].

The literature also reports sedimentation coefficients of 39S for the aggregation form 4 × hexamer (4 × 6) of Hcs from crustaceans, with aggregation states in the 25S and 29S transition range [36,45,47]. However, the present study observed no species with sedimentation values lower or higher than 19S (Figure 6A), indicating a high homogeneity in a solution of a single Hc species. These results are consistent with those previously reported for crustacean hemocyanins in the hexameric (1 × 6) form, which exhibit a sedimentation coefficient of approximately 17S [45,48].

According to the literature, crustacean Hcs comprise six structural and functional monomeric subunits (with MM around 75 kDa) with a di-nuclear Cu center that reversibly binds to O2. They are found as oligomers in the complex hexamer form [36,45].

Hc from the Norway lobster (*Nephrops norvegicus*) shows monomeric subunits with MM close to 72 and 74 kDa on SDS-PAGE gels. By dynamic light scattering (DLS) analyses, the purified native Hc from the crustacean showed a spin radius of 8.3 ± 2 nm, suggesting that the Hc was present as a hexamer. The data presented indicate that the HcMac oligomeric structure comprises 75 and 76 kDa monomeric chains, which combine to create a 1 × hexamer with a mass of 450 kDa.

Investigations into the solvent type, pH, and ionic strength are vital for comprehending the principal factors that impact the stability of hemoproteins. pH values represent the equilibrium between H^+^ and OH^−^ ions in a solution, and any alteration affects the ionization equilibrium of acidic and alkaline groups in a protein. Thus, fluctuations in pH affect the protein’s charge distribution, leading to modifications in electrostatic interactions between protein subunits, between the protein and the solvent, and between solvent molecules. These changes can cause conformational variations in the protein structure, enabling the assessment of pH-induced alterations in oligomeric stability, protein folding, and function [49,50].

In Figure 7A, the absorption spectrum of HcMac exhibits a band at 280 nm related to the aromatic amino acids and another at 340 nm associated with the di-copper center. These findings are consistent with the typical absorption spectrum of other oxy-Hcs [12,46].

The results indicate that HcMac has a greater affinity for oxygen at pH levels close to 4.4 and above 8.0 when maintained at 25 °C. This behavior may be linked to the protein’s unfolding at alkaline pH levels and initiating the acid denaturation process when pH levels drop below 4.9. On the other hand, molluscan multimeric oxy-Hc displays a limited range of reversibility of acid denaturation at pH values greater than 5.0, resulting in a stronger binding affinity to oxygen [24].

In a comparative study of Hc from five species of mud shrimp, ref. [45] observed that the Hcs exhibited greater affinity for oxygen in the pH range of 7.68 to 7.83 at a temperature of 10 °C. The Hc of the ghost shrimp, *Callianassa californiensis*, displayed the highest oxygen affinity at pH 8.2 and at a temperature of 15 °C [12]. In contrast, the hemocyanin of the crab *Bythograea thermydron* exhibited the highest oxygen affinity at pH 8.0 and 15 °C [51].

The results of monitoring Trp residue through fluorescence emission after excitation at 295 nm indicate that the oxy-HcMac form at pH 7.0 displays a fluorescence maximum at 327 ± 1 nm (Figure 7). This suggests that the Trp residue is located in a non-polar environment. [50,52,53] examined changes in the microenvironment of Trp residues in the Hc of *Megathura crenulata*, commonly known as Keyhole limpet hemocyanin (KLH), through variations in Trp fluorescence intensity and λ_max_ as a function of pH. Between pH 4.0 and 7.4, no changes in fluorescence intensity at 340 nm or λ_max_ were observed. However, when pH was reduced to below 4.0, at 2.8, the fluorescence intensity increased substantially, and there was a shift in λ_max_ towards higher values from 332 to 344 nm. The authors believed that this shift in λ_max_ indicates an increase in polarity in the microenvironment of the Trp residue. The rise in fluorescence intensity during the unfolding of KLH under acidic conditions may be due to the reduction in suppression of some Trp residues.

The hemocyanin (Hc) from the garden snail *Helix lucorum* (HlH) and its functional subunits (FU) were studied using fluorescence spectroscopy, with excitation at 295 nm to monitor the tryptophan side chains. The fluorescence of HlH exhibited a λ_max_ of 335 ± 1 nm, which indicates that Trp is deeply buried in a hydrophobic environment. In contrast, the λ_max_ of FU was shifted to 342 ± 1 nm due to the exposure of Trp side chains on the molecule’s surface [54]. The exposure of Trp residues promotes energy transfer to copper (Cu), a heavy metal ion coordinated at the protein’s active site, thereby influencing the observed fluorescence emission profile.

The increase in fluorescence intensity observed in hemoproteins is consistent with reduced energy transfer from tryptophan residues to the heme group, where greater exposure of fluorophores to the aqueous solvent may be associated with protein dissociation [46].

The decrease in fluorescence intensity, especially above pH 7.4, may be attributed to the dissociation of HcMac (Figure 8). When the protein dissociates, the Trp residues are exposed to the solvent and may undergo fluorescence suppression due to their proximity to the heavy metal of the di-copper center. It is worth noting that hemocyanins typically contain about 5 to 8 Trp residues per subunit [46].

According to the findings of [24], electron microscopy revealed that pH 9.6 induces a strong dissociation environment for Hc from the mollusk *Cornu aspersum* (CaH), resulting in the prevalence of isolated and/or compact monomeric CaH subunits. The data indicate that under these conditions, the CaH subunits do not dissociate any further.

The LSI measurements of HcMac resulted in two distinct plateaus of greater intensity. The first plateau appears within the pH range of 4.2 to 5.3, which may be attributed to the isoelectric point (*pI*) of HcMac in the region with acidic pH values, as observed in other crustacean hemocyanins with acidic *pI* [39,55,56].

Crustacean Hc studies have reported variations in the *pI* values. For instance, the hemocyanin of the white shrimp *Penaeus vannamei* displays two bands in the two-dimensional electrophoresis gel, with *pI* values of approximately 4.8 and 4.9 [39]. Another shrimp species, *Penaeus indicus*, exhibits two *pI* values of 4.2 and 4.25 [56]. The hemocyanin of the lobster *Palunirus japonicus* comprises four subunits with varying *pI* values, such as 79 kDa with *pI* = 5.38, 84 kDa with *pI* = 5.06, 88 kDa with *pI* = 5.11, and 90 kDa with *pI* = 5.1 [55].

The second plateau in the LSI measurements between pH 9.3 and 10.5, as shown in Figure 9, may indicate the aggregation or denaturation of HcMac. This observation is consistent with the decrease in fluorescence emission intensity (Figure 8B) and the increase in absorption at 340 nm (Figure 7C) due to the higher exposure of the di-copper center and the presence of O_2_.

The spectroscopic data suggest that HcMac has higher stability within the pH range of 5.5–7.4. UV-Vis measurements indicate that the di-copper center has the highest affinity for O_2_ within this range (Figure 7C). There are minimal changes in the fluorescence emission intensity and λ_max_ emission (shown in Figure 8B and Figure 8C, respectively). Furthermore, according to the LSI data, there are negligible alterations in the oligomer size. The findings are comparable to those reported by [52], who observed, through circular dichroism (CD) analysis, that the KLH molecule did not undergo significant changes in ellipticity at 222 nm within the pH range of 5.0 to 8.0, except for a small interval of partial reversibility in the acidic medium. Below pH 5.0, there was a significant reduction, with the lowest value recorded at pH 2.8.

## 5. Conclusions

The biophysical characterization of hemocyanin from *Macrobrachium acanthurus* (HcMac) in this study revealed key structural and functional properties, highlighting its potential for biotechnological applications. Spectroscopic analysis demonstrated that HcMac maintains its oligomeric stability within the pH range of 5.0–7.4, with the highest oxygen affinity observed in this interval. In contrast, extreme pH variations induce significant conformational changes. These findings align with previous studies on crustacean Hemocyanins (Hcs), indicating that structural stability and oxygen transport capacity are highly pH-dependent.

Furthermore, Analytical Ultracentrifugation Analysis (AUC) and fluorescence spectroscopy data confirmed that HcMac consists of monomeric subunits of 75 and 76 kDa, forming a 450 kDa hexamer, similar to other crustacean Hcs. The absence of alternative oligomeric species in solution suggests high structural homogeneity, which may be a key factor for its biological functionality and biotechnological applicability.

The relevance of these findings extends to potential biomedical and industrial applications, as Hcs from various species have demonstrated antimicrobial and immunomodulatory activity as well as promising structural properties for the development of biopharmaceuticals and vaccine adjuvants. Given the growing demand for new therapeutic strategies and the search for stable and bioactive proteins, the presented data reinforce HcMac as a promising candidate for further research in biotechnology and healthcare.

Future investigations should explore the biological functionality of this protein in greater depth, as well as its interactions with immune systems and potential application in the biotechnological industry. Integrating advanced bioinformatics, proteomics, and immunology approaches will be essential to expand the understanding of HcMac and maximize its utilization in novel therapeutic and industrial technologies.

## Figures and Tables

**Table 1:** Biotechnological and medical applications of crustacean and mollusk hemocyanins.

Summarizes Investigations for		
Applications	Study Animal Species	References
Biomarkers of environmental pollution	Red swamp crayfish (*Procambarus clarki*); European green crab (*Carcinus maenas*); American lobster (*Homarus americanus*); Shrimp (*Pandalus platyceros*).	[27,31]
Molecular evolution research	European lobster (*Homarus gammarus*); Green crab (*Carcinus maenas*); Giant tiger shrimp (*Penaeus monodon*).	[28]
Immunological adjuvants	White-legged shrimp (*Litopenaeus vannamei*)	[32]
Cancer treatment	Common snail (*Helix aspersa*); Turkish snail (*Helix lucorum*); Rapana shell (*Rapana venosa*).	[29]

## Data Availability

The original contributions presented in this study are included in the article. Further inquiries can be directed to the corresponding author.

## Supplementary Materials

The following supporting information can be downloaded at: https://www.mdpi.com/article/10.3390/biom15050675/s1, Figure S1: Original Western blot images of Figure 1.

## References

[1] Huang Y., Ren Q. (2021). Innate immune responses against viral pathogens in Macrobrachium. Dev. Comp. Immunol..

[2] Coates C.J., Nairn J. (2014). Diverse immune functions of hemocyanins. Dev. Comp. Immunol..

[3] Todorovska E., Ivanov M., Radkova M., Dolashki A., Dolashka P. (2021). Molecular Cloning, Structure and Phylogenetic Analysis of a Hemocyanin Subunit from the Black Sea Crustacean *Eriphia verrucosa* (Crustacea, Malacostraca). Genes.

[4] Adachi K., Hirata T., Nishioka T., Sakaguchi M. (2003). Hemocyte components in crustaceans convert hemocyanin into a phenoloxidase-like enzyme. Comp. Biochem. Physiol. Part B Biochem. Mol. Biol..

[5] Gianazza E., Eberini I., Palazzolo L., Miller I. (2021). Hemolymph proteins: An overview across marine arthropods and molluscs. J. Proteom..

[6] Dilna C., Prasanth G.K., Ghufran M.S., Soni P., Kanade S.R., Duddukuri G.R. (2023). Purification and characterization of a hemocyanin with lectin-like activity isolated from the hemolymph of speckled shrimp, *Metapenaeus monoceros*. Biochimie.

[7] García-Carreño F.L., Cota K., Navarrete Del Toro M.A. (2008). Phenoloxidase Activity of Hemocyanin in Whiteleg Shrimp Penaeus vannamei: Conversion, Characterization of Catalytic Properties, and Role in Postmortem Melanosis. J. Agric. Food Chem..

[8] Mendoza-Porras O., Kamath S., Harris J.O., Colgrave M.L., Huerlimann R., Lopata A.L., Wade N.M. (2020). Resolving hemocyanin isoform complexity in haemolymph of black tiger shrimp Penaeus monodon—Implications in aquaculture, medicine and food safety. J. Proteom..

[9] Li H., Xia X., Wang Z., Zang J., Du M. (2023). Oyster (*Crassostrea gigas*) ferritin should be a promising Fe2+ nanocarrier. Food Chem..

[10] Coates C.J., Decker H. (2017). Immunological properties of oxygen-transport proteins: Hemoglobin, hemocyanin and hemerythrin. Cell. Mol. Life Sci..

[11] Marxen J.C., Pick C., Kwiatkowski M., Burmester T. (2013). Molecular characterization and evolution of haemocyanin from the two freshwater shrimps *Caridina multidentata* (Stimpson, 1860) and *Atyopsis moluccensis* (De Haan, 1849). J. Comp. Physiol. B.

[12] Coates C.J., Nairn J. (2013). Hemocyanin-derived phenoloxidase activity: A contributing factor to hyperpigmentation in *Nephrops norvegicus*. Food Chem..

[13] Ji R., Guan L., Hu Z., Cheng Y., Cai M., Zhao G., Zang J. (2024). A comprehensive review on hemocyanin from marine products: Structure, functions, its implications for the food industry and beyond. Int. J. Biol. Macromol..

[14] Masuda T., Baba S., Matsuo K., Ito S., Mikami B. (2020). The high-resolution crystal structure of lobster hemocyanin shows its enzymatic capability as a phenoloxidase. Arch. Biochem. Biophys..

[15] Markl J. (2013). Evolution of molluscan hemocyanin structures. Biochim. Biophys. Acta (BBA) Proteins Proteom..

[16] Lieb B., Dimitrova K., Kang H.S., Braun S., Gebauer W., Martin A., Hanelt B., Saenz S.A., Adema C.M., Mark J. (2006). Red blood with blue-blood ancestry: Intriguing structure of a snail hemoglobin. Proc. Natl. Acad. Sci. USA.

[17] Van Holde K.E., Miller K.I., Decker H. (2001). Hemocyanins and Invertebrate Evolution. J. Biol. Chem..

[18] Zhao M., Zheng Z., Wang C., Yao D., Lin Z., Zhao Y., Chen X., Li S., Aweya J.J., Zhang Y. (2023). Penaeid shrimp counteract high ammonia stress by generating and using functional peptides from hemocyanin, such as HMCs27. Sci. Total Environ..

[19] Tassanakajon A., Rimphanitchayakit V., Visetnan S., Amparyup P., Somboonwiwat K., Charoensapsri W., Tang S. (2018). Shrimp humoral responses against pathogens: Antimicrobial peptides and melanization. Dev. Comp. Immunol..

[20] Cerenius L., Söderhäll K. (2021). Immune properties of invertebrate phenoloxidases. Dev. Comp. Immunol..

[21] Liu S., Aweya J.J., Zheng L., Wang F., Zheng Z., Zhong M., Lun J., Zhang Y. (2018). A Litopenaeus vannamei Hemocyanin-Derived Antimicrobial Peptide (Peptide B11) Attenuates Cancer Cells’ Proliferation. Molecules.

[22] Kizheva Y.K., Rasheva I.K., Petrova M.N., Milosheva-Ivanova A.V., Velkova L.G., Dolashka P.A., Dolashki A.K., Hristova P.K. (2019). Antibacterial activity of crab haemocyanin against clinical pathogens. Biotechnol. Biotechnol. Equip..

[23] Petrova M., Vlahova Z., Schröder M., Todorova J., Tzintzarov A., Gospodinov A., Velkova L., Kaynarov D., Dolashki A., Dolashka P. (2023). Antitumor Activity of Bioactive Compounds from Rapana venosa against Human Breast Cell Lines. Pharmaceuticals.

[24] Dolashki A., Velkova L., Voelter W., Dolashka P. (2019). Structural and conformational stability of hemocyanin from the garden snail *Cornu aspersum*. Z. Für Naturforschung C.

[25] Lyu X., Tsui Y.M., Ho D.W.H., Ng I.O.L. (2022). Liquid Biopsy Using Cell-Free or Circulating Tumor DNA in the Management of Hepatocellular Carcinoma. Cell. Mol. Gastroenterol. Hepatol..

[26] Alinejad T., Bin K.Q., Vejayan J., Othman R.Y., Bhassu S. (2015). Proteomic analysis of differentially expressed protein in hemocytes of wild giant freshwater prawn Macrobrachium rosenbergii infected with infectious hypodermal and hematopoietic necrosis virus (IHHNV). Meta Gene.

[27] Engel D., Brouwer M., McKenna S. (1993). Hemocyanin concentrations in marine crustaceans as a function of environmental conditions. Mar. Ecol. Prog. Ser..

[28] Toon A., Finley M., Staples J., Crandall K., Martin J., Crandall K., Felder D. (2009). Decapod Phylogenetics and Molecular Evolution. Decapod Crustacean Phylogenetics.

[29] Georgieva A., Todorova K., Iliev I., Dilcheva V., Vladov I., Petkova S., Dolashki A., Velkova L., Dolashka P., Toshkova R. (2023). Assessment of the In Vitro and In Vivo Antitumor Activity of Hemocyanins from Helix aspersa, Helix lucorum, and Rapana venosa in a Graffi Myeloid Tumor Model. Biomedicines.

[30] Harnedy P.A., FitzGerald R.J. (2012). Bioactive peptides from marine processing waste and shellfish: A review. J. Funct. Foods.

[31] Wei K., Yang J. (2015). Oxidative damage of hepatopancreas induced by pollution depresses humoral immunity response in the freshwater crayfish Procambarus clarkii. Fish Shellfish. Immunol..

[32] Wang Q., Liu N., Wang J.X., Wu Y.L., Wang L. (2014). Physiological changes and acetylcholinesterase activity in the cladoceran Moina macrocopa (Straus, 1820) exposed to mercury and sodium dodecyl sulfate. Crustaceana.

[33] Anger K. (2013). Neotropical Macrobrachium (Caridea: Palaemonidae): On the biology, origin, and radiation of freshwater-invading shrimp. J. Crustac. Biol..

[34] Pileggi L., Rossi N., Wehrtmann I., Mantelatto F. (2014). Molecular perspective on the American transisthmian species of Macrobrachium (Caridea, Palaemonidae). ZooKeys.

[35] Huang Y., Tan D., Chen X., Xia B., Zhao Y., Chen X., Zhang Y., Zheng Z. (2024). Function of hemocyanin-mediated succinate dehydrogenase in glucose metabolism and immunity of Penaeus vannamei. Fish Shellfish. Immunol..

[36] Paoli M., Giomi F., Hellmann N., Jaenicke E., Decker H., Di Muro P., Beltramini M. (2007). The molecular heterogeneity of hemocyanin: Structural and functional properties of the 4 × 6-meric protein of Upogebia pusilla (Crustacea). Gene.

[37] Carvalho F.A.O., Carvalho J.W.P., Santiago P.S., Tabak M. (2011). Further characterization of the subunits of the giant extracellular hemoglobin of Glossoscolex paulistus (HbGp) by SDS-PAGE electrophoresis and MALDI-TOF-MS. Process Biochem..

[38] Schuck P. (2003). On the analysis of protein self-association by sedimentation velocity analytical ultracentrifugation. Anal. Biochem..

[39] Figueroa-Soto C.G., De La Barca A.M.C., Vazquez-Moreno L., Higuera-Ciapara I., Yepiz-Plascencia G. (1997). Purification of Hemocyanin from White Shrimp (Penaeus vannamei Boone) by Immobilized Metal Affinity Chromatography. Comp. Biochem. Physiol. Part B Biochem. Mol. Biol..

[40] Mullaivanam Ramasamy S., Denis M., Sivakumar S., Munusamy A. (2017). Phenoloxidase activity in humoral plasma, hemocyanin and hemocyanin separated proteins of the giant freshwater prawn *Macrobrachium rosenbergii*. Int. J. Biol. Macromol..

[41] De Oliveira A.M., Malunga L.N., Perussello C.A., Beta T., Ribani R.H. (2020). Phenolic acids from fruits of *Physalis angulata* L. in two stages of maturation. South Afr. J. Bot..

[42] Jaenicke E., Decker H. (2003). Tyrosinases from crustaceans form hexamers. Biochem. J..

[43] Lee S.Y., Lee B.L., Söderhäll K. (2004). Processing of crayfish hemocyanin subunits into phenoloxidase. Biochem. Biophys. Res. Commun..

[44] Wright J., Clark W.M., Cain J.A., Patterson A., Coates C.J., Nairn J. (2012). Effects of known phenoloxidase inhibitors on hemocyanin-derived phenoloxidase from Limulus polyphemus. Comp. Biochem. Physiol. Part B Biochem. Mol. Biol..

[45] Taylor A.C., Astall C.M., Atkinson R.J.A. (2000). A comparative study of the oxygen transporting properties of the haemocyanin of five species of thalassinidean mud-shrimps. J. Exp. Mar. Biol. Ecol..

[46] Baird S., Kelly S.M., Price N.C., Jaenicke E., Meesters C., Nillius D., Decker H., Nairn J. (2007). Hemocyanin conformational changes associated with SDS-induced phenol oxidase activation. Biochim. Biophys. Acta (BBA) Proteins Proteom..

[47] Arisaka F., Van Holde K.E. (1979). Allosteric properties and the association equilibria of hemocyanin from Callianassa californiensis. J. Mol. Biol..

[48] Miller K.I., Eldred N.W., Arisaka F., Van Holde K.E. (1977). Structure and function of hemocyanin from thalassinid shrimp. J. Comp. Physiol. B.

[49] Santiago P.S., Moura F., Moreira L.M., Domingues M.M., Santos N.C., Tabak M. (2008). Dynamic Light Scattering and Optical Absorption Spectroscopy Study of pH and Temperature Stabilities of the Extracellular Hemoglobin of *Glossoscolex paulistus*. Biophys. J..

[50] Raynova Y., Angelov I., Idakieva K. (2020). Fluorescence Properties and Conformational Stability of Hemocyanin Isolated from Snails Helix Aspersa Maxima. J. Chem. Technol. Metall..

[51] Sanders N.K., Childress J.J. (1992). Specific effects of thiosulphate and L-lactate on hemocyanin-O2 affinity in a brachyuran hydrothermal vent crab. Mar. Biol..

[52] Varshney A., Ahmad B., Rabbani G., Kumar V., Yadav S., Khan R.H. (2010). Acid-induced unfolding of didecameric keyhole limpet hemocyanin: Detection and characterizations of decameric and tetrameric intermediate states. Amino Acids.

[53] Idakieva K., Siddiqui N.I., Parvanova K., Nikolov P., Gielens C. (2006). Fluorescence properties and conformational stability of the β-hemocyanin of *Helix pomatia*. Biochim. Biophys. Acta (BBA) Proteins Proteom..

[54] Velkova L., Dimitrov I., Schwarz H., Stevanovic S., Voelter W., Salvato B., Dolashka-Angelova P. (2010). Structure of hemocyanin from garden snail *Helix lucorum*. Comp. Biochem. Physiol. Part B Biochem. Mol. Biol..

[55] Makino N., Kimura S. (1988). Subunits of *Panulirus japonicus* hemocyanin: 1. Isolation and properties. Eur. J. Biochem..

[56] Noel P.Y., Martin M. (1995). Comparative Study of Hemocyanins of Decapoda Using Isoelectric Focusing. J. Crustac. Biol..

