# Supplementary material for: Biophysical Characterization of Shrimp Hemocyanins: Stability and Emerging Biotechnological Applications
Source: Biomolecules. 2025 May 6;15(5):675. doi: 10.3390/biom15050675 (PMC12108899; doi:10.3390/biom15050675)
Supplement: Supplementary file 1 [file biomolecules-15-00675-s001.zip › biomolecules-3509801-supplementary.pdf]

Supplementary Materials

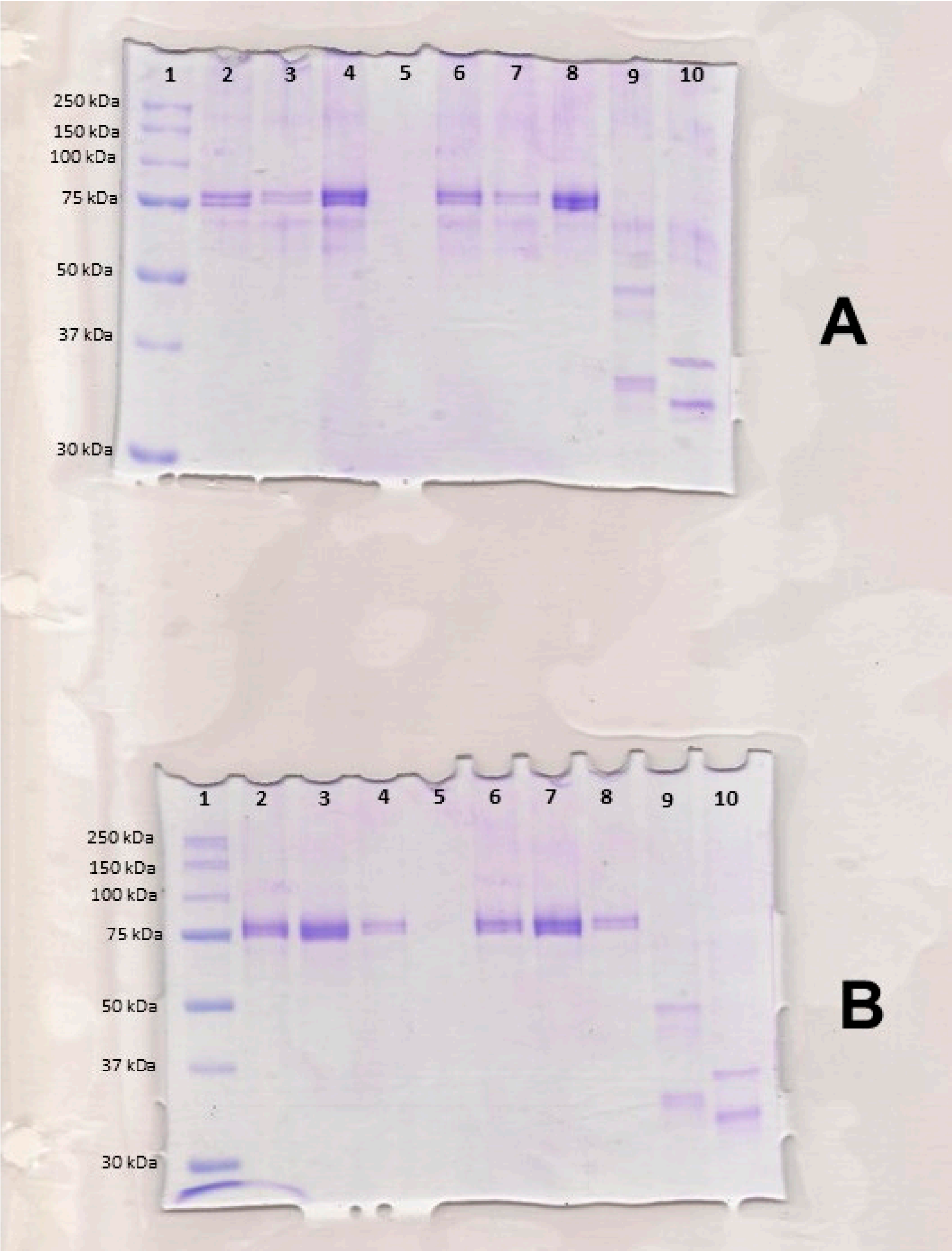

Figure S1. Original Western blot images of Figure 1. (**A**) SDS PAGE electrophoresis gel (10% acrylamide, *v*/*v*) prepared without the addition of the reducing agent β-mercaptoethanol. The wells are numbered 1 to 10. Well 1: molecular weight standard; Well 2: 15 µL aliquot of HcMac; Well 3: 10 µL aliquot of HcMac; Well 4: 5 µL aliquot of HcMac; Well 5: empty; Well 6: 15 µL aliquot of HcMac; Well 7: 10 µL aliquot of HcMac; and Well 8: 5 µL aliquot of HcMac. In wells 9 and 10, 10 µL aliquots of hemoglobin from the annelids *Glossoscolex paulistus* and *Amynthas gracilis* were added to monitor the dissociation of HcMac; (**B**) Gel prepared with the addition of the reducing agent β-mercaptoethanol. The wells were filled with the same sequence as in (**A**).
